# The Relationship between Restrictions on Going Out and Motor Imagery among Medical University Students in Japan—Research with Small Samples

**DOI:** 10.3390/life13030797

**Published:** 2023-03-15

**Authors:** Keisuke Itotani, Ippei Suganuma, Seiji Morimoto, Hideaki Nakai, Noriyuki Ogawa

**Affiliations:** 1 Department of General Rehabilitation, Faculty of Allied Health Sciences, Yamato University, 2-5-1 Katayama-cho, Suita, Osaka 564-0082, Japan; 2 Department of Occupational Therapy, Faculty of Health Sciences, Kyoto Tachibana University, 34 Oyakeyamada-cho, Yamashina-ku, Kyoto 607-8175, Japan; 3 Department of Occupational Therapy, Faculty of Health Sciences, Aino University, 4-5-4 Higashiota, Osaka 567-0012, Japan

**Keywords:** restrictions on going out, imagined Timed Up and Go test, motor imagery

## Abstract

Motor imagery is often used as a training method to improve physical performance. Previous studies have often reported that reduced motor imagery is more likely to occur in older adults and stroke patients. However, it has also been reported that it is difficult to imagine exercises that cannot be performed. Therefore, we hypothesized that this may also have occurred in young people who were physically able to exercise but who were restricted by COVID-19 lockdowns, however, we could find no studies that investigated the impact of restricting outings. In this study, 83 healthy young people were measured for physical performance (maximum walking speed, grasp strength, Timed Up and Go test, imagined Timed Up and Go test, functional reach test, and five chair stand test). It was found that, while restricting outings did not influence physical performance in the subjects, it did influence motor imagery. Therefore, it should be borne in mind that training with motor imagery may not generate adequate actual motor imagery when restrictions are imposed on activities.

## 1. Introduction

Over the past few years, Japan has experienced a decline in physical activity due to the effects of COVID-19 [1]. The impact was particularly noticeable from 2019 to 2021. In that period, the Japanese government requested the public to limit unnecessary and non-urgent outings. The term ‘lockdown’ was not used, but, rather, ‘restrictions on going out’ were strongly requested under regional ‘States of Emergency’. Such requests were made if a rapid increase in the number of infected people was recognized on a daily basis and were preventative measures to counter the spread of COVID-19. As a result, teleworking was introduced to working people and remote lectures were implemented in schools, and the number of workplaces and education facilities (elementary schools, junior high schools, high schools, universities, etc.) that accepted the measures increased rapidly. Due to strong requests from the national government and local governments, many people were forced to stay at home, and it was reported that physical activity decreased [1]. Furthermore, according to a survey conducted by Health Japan 21 in Japan, the percentage of people aged 20 to 64 who exercise regularly (those who exercised for 30 min or more a day at least twice a week for at least one year) was 24.6% in 2010 and 21.5% in 2016 [2], and, while an increase of various means of transportation and support makes daily life easier, the amount of physical activity among young people is decreasing. From the above, it can be said that young people who have been affected by restrictions on going out have decreased physical activity. Decreased physical activity among young people not only increases the risk of lifestyle-related diseases, but also has adverse health effects such as obesity, muscle atrophy, and bone loss due to a lack of exercise [3]. Moreover, it is a known fact that these cause a decline in exercise performance. However, while it is known that confinement exacerbates frailty and weakens the immune system in older adults [4,5], restricting exercise for young people has received little attention.

On the other hand, there are training methods that improve exercise performance without physical activity. One such method uses motor imagery and recalls one’s own exercise. Jeannerod [6] concludes that motor imagery is functionally equivalent to motor planning that occurs in the unconscious pre-movement context and intentional pre-movement motor imagery, as they share common mechanisms. In previous studies using motor imagery, one study found an improvement of one-legged standing balance after intervention using motor imagery in older adult women [7], and another study focused on the improvement of muscle strength through motor imagery training without actually causing muscle activity [8]. Therefore, by training before exercise with an image of the exercise to actually be performed, an improvement in exercise performance may be recognized. Furthermore, it has been confirmed that the walking speed of hemiplegic stroke patients is improved [9] and the flexibility of healthy subjects is improved [10].

However, it has also been stated that, although training using motor imagery im-proves motor performance, imagery training for exercises that cannot subsequentially be performed is ineffective [11]. Therefore, if the amount of physical activity decreases due to restrictions on going out, it may not be possible to perform exercise imagery appropriately. If motor imagery can be performed appropriately even when going out is restricted, it may be possible to show the effectiveness of training using motor imagery within the range of daily living activities at home.

In this study, it was decided to investigate whether restrictions on physical activity in restrictions on going out are a factor for performing appropriate motor imagery. If motor imagery cannot be appropriately recalled due to a decrease in physical activity, training using motor imagery [7,8,9,10] must always assess the subject’s physical activity. Conversely, if the recollection of appropriate motor imagery is not affected by the amount of physical activity, the motor imagery can be used more effectively.

## 2. Materials and Methods

The subjects were 83 university students who attended remote lectures in 2019 (those who took remote lectures at home) due to non-essential outing restrictions, or who at-tended regular face-to-face lectures in 2020 (those who attended lectures at universities). The period of remote lectures due to requests for restrictions on going out was about two months, in September and October 2020. The area where the subjects go to university has three different national highways, and a railway station and airport are located within 10 km of the city center. In addition, national highways, arterial roads, and several railway lines pass through the area, allowing for smooth travel to and from the city center and neighboring cities. Exclusion criteria followed previous studies [12], excluding those with orthopedic problems (fractures, osteoarthritis, ligamentous injuries, pain, paresthesia, etc.) or neurological abnormalities. The subjects were classified into two groups: the restriction of outings group (R-group) were students who had remote lectures due to restrictions on going out in 2019, and the non-restriction of outing group (NR-group) were students who had regular face-to-face lectures in 2020. Information about this study was provided in writing to all subjects prior to starting the assessment, and all subjects provided their informed consent. This study was approved by the Research Ethics Committee of the Faculty of Health Sciences, Yamato University.

### 2.1. Characteristics and Measurements of Physical Performance

Table 1 shows the characteristics of the subjects. These were height, weight, BMI, sex, grade point average (GPA), and primary means of commuting to university (walking, bi-cycle, motorcycle, train, other). Subjects’ academic performance was based on GPA [13]. This academic performance is graded on a four-point scale for each subject. Four-level classification: 100–90 score = 4 points, 89–80 score = 3 points, 79–70 score = 2 points, and 69–60 score = 1 point. Overall, a GPA estimated with required subjects (GPA-RS) was calculated. Measurements of physical performance were maximum walking speed (MWS), Timed Up and Go test (TUG), imagined TUG (iTUG), functional reach test (FRT), 5 chair stand (5CS), and grasp strength. MWS [12], TUG [14], 5CS [15], FRT [16], and grasp strength [17] were measured with reference to previous studies [12,14,15,16,17].

In MWS, a stopwatch was used. Measurements started and ended when the subject’s torso reached the start and end of the measurement section. The measurement section was 5 m with a 1 m preliminary passage before and after the section. Each subject was instructed, “Walk fast, but don’t run”. Speed was measured twice, averaged, and then converted into a speed (m/s).

In TUG, the time was measured using a stopwatch, from standing up from a chair with a seat height of about 45 cm with a backrest, walking around a cone 3 m away, and sitting down again. The measurement was taken from the signal of ‘ready go’ to the subject sitting back down in the chair and at a comfortable walking speed.

iTUG was measured using a stopwatch according to previous studies [18,19], and this measurement was always performed before measuring TUG. Firstly, subjects sat in a chair and imagined performing TUG. In other words, subjects imaged ‘stand up from a chair, walk around a cone 3 m away, and sit down again on the chair’. Subjects could choose to do iTUG with their eyes open or closed. The stopwatch measurement was started with the measurer’s ‘ready go’ and stopped when the subjects said ‘stop’. The subject’s image of standing up from a chair was started by the measurer’s call of ‘ready go’. Subjects called ‘stop’ when they were ‘sitting down again’ in their image and were instructed to say so loudly. Subjects were asked to perform iTUG and TUG at a normal speed of their own choosing. iTUG was performed only once, followed by TUG, also performed once. Both times were recorded with a stopwatch to the nearest 0.01 s. From TUG and iTUG, delta time was calculated based on previous research [18]; [(TUG − iTUG)/(TUG + iTUG)/2)] × 100.

In 5CS, a stopwatch and a chair with a seat height of about 45 cm with a backrest were used. The subjects were asked to quickly stand up and sit down five times in succession from a sitting position, and the time was measured. The starting position was sitting, and the end position was the static standing position after they stood up for the fifth time. During the measurement, the subjects crossed both arms in front of their chest. Furthermore, it was confirmed that both knee joints were fully extended in the standing position, and that the gluteal were always in contact with the seat surface each time in the sitting position.

In FRT, first, the shoulder joint on the measurement side was flexed 90° to the finger extension, elbow joint extension, and mid-forearm position. After that, a standing position was maintained so that the upper extremities were parallel to the wall. The wall was marked with the position of the tip of the middle finger of the upper limb on the measurement side. The upper limb was then reached forward as far as possible and the position of the tip of the middle finger was marked again. A perpendicular line to the floor was drawn at both the start marking point and the marking point when reached, the distance (cm) of which was measured. If they moved their feet or any part of their body contacted the wall, they were remeasured.

Grasp strength was measured by a Smedley-Type Hand Dynamometer Grip D (Takei Kiki Kogyo, Tokyo, Japan). Measurements were taken twice on the dominant hand, and the maximum value was used for analysis.

### 2.2. Statistical Analysis

Height, weight, BMI, GPA, MWS, TUG, iTUG, delta time, FRT, 5CS, and grasp strength outcome variables were mean ± standard deviation. Sex and means of commuting to university variables were the number of people. These variables were analyzed using a Student’s *t*-test and chi-square test. Normality was checked using the Shapiro–Wilk normality test. Furthermore, as previous studies [20] have reported that iTUG was affected by sex, we tested the interaction between the two factors using a two-way ANOVA, with the presence or absence of restrictions on going out (R-group/NR-group) and sex (male/female) as two factors and calculated the effect size (η^2^). The significance level was set at *p* < 0.05. Analysis was performed using IBM SPSS version 26 (IBM Corporation, Armonk, NY, USA).

## 3. Results

Of the 83 subjects, 42 were in the R-group and 41 were in the NR-group. In both groups, most of the students used the railway as their primary means of commuting to university. As a result of the Student’s *t*-test and chi-square test for subject attributes, no significant difference was observed in any variable (Table 2). Comparisons between the two groups in the Student’s *t*-test showed that, for physical performance, the R-group had a significantly shorter iTUG than the NR-group (R-group vs. NR-group; 5.8 ± 1.4 vs. 7.4 ± 1.6, *p* < 0.001, r = 0.48), and ΔTUG was significantly higher (R-group vs. NR-group; 15.4 ± 16.3 vs. 1.7 ± 10.9, *p* < 0.001, r = 0.43). On the other hand, no significant differences were found in MWS, FRT, grasp strength, or 5CS. Furthermore, a two-way ANOVA with the presence or absence of restrictions on going out (R-group/NR-group) and sex (male/female) as two factors is shown in Table 3. The results showed that there was no interaction between whether or not a person was restricted in going out and sex in ΔTUG, only a significant difference in the main effect of restricting going out (*p* < 0.001, η^2^ = 0.19).

## 4. Discussion

The present study examined the effect of restrictions on going out on motor imagery in medical university students in one urban area in Japan. The results showed that the R-group had significantly shorter iTUG and higher ΔTUG than the NR-group. No significant differences were found in walking speed, balance, or upper and lower limb muscle strength. From these, it was shown that there is a high possibility that restrictions on going out have an effect on motor imagery. However, there was no significant difference in walking ability, balance ability, or lower limb muscle strength between the R-group and the NR-group. Therefore, walking and balance ability and leg muscle strength are considered to be less affected by restrictions on going out for young people. In addition, the two-way ANOVA showed no interaction between restrictions on going out and sex in ΔTUG. This result is different from the results of a previous study [20], which showed that ΔTUG was affected by sex, and it became clear that only restrictions on going out had an effect. Decreased walking speed is found in older adults with cognitive decline [21], stroke patients [22], and those with Parkinson’s disease [23], but the subjects of this study were young people with no cognitive decline or disease. Leg muscle weakness [24,25,26], lower limbs joint impairment [25], and poor balance ability [26] have been reported to occur, but, in this study, there were no significant differences between the R-group and NR-group in 5CS, FRT, or TUG. Since no such findings were observed, it was considered that the two groups had similar levels of leg muscle strength, flexibility, and balance ability. In grasp strength, upper limb muscle mass is less likely to decrease than lower limb mass and trunk muscle mass [27], and a longitudinal study [28] reported a significant decrease from around 60 years of age, compared to a baseline of 40 years of age. From these, it seems that the NR-group in this study had the same level of grasp strength as the R-group, although they had restrictions on going out. Furthermore, in the 5CS, the muscle strength of the lower limbs was more likely to decrease than that of the upper limbs [27], but no significant difference was shown between the NR-group and the R-group. It has been shown that lower limb muscle strength decreases by approximately 28%, even in young people, if immobility continues for two weeks [29]. However, in this study, although the R-group was restricted from going out, there was no restriction on ADL at home, so it is considered that there was no significant difference in the NR-group compared to the R-group. It has also been shown that Type I fibers (slow muscles) are more likely to be reduced than Type II (fast muscles) in the case of inactivity [30]. From these results, it also appears that the NR-group and R-group had similar muscle strength, as grasp strength and 5CS in the present study require Type II fast muscle strength. The FRT results also indicate that the hamstring flexibility of both groups is also comparable, so it is likely that no difference was found between the two groups in MWS, which is affected by lower limb muscle strength and hamstring flexibility from previous studies [12]. 

For motor imagery, iTUG has been used by previous studies [18], and the accuracy of motor imagery is assessed with ΔTUG. ΔTUG has been reported to decrease in older adults [18] and stroke patients [31]. Motor imagery is influenced by the frontal lobe, which is responsible for planning movement. For this reason, older adults who are prone to frontal lobe atrophy [18] and stroke patients who have suffered brain damage have difficulty generating motor imagery appropriately due to declined cognitive function [31]. However, the subjects in this study were healthy young people, and no significant differences were shown between the two groups in GPA-RS, which indicates academic performance. Therefore, it is thought that restrictions on going out affect ΔTUG in young people, and there is a high possibility that restrictions on going out may have decreased the motor imagery in university students. 

The limitations of this study are that it was not possible to compare the same group before and after the implementation of restrictions on going out, and that it was not possible to objectively measure how much physical activity was limited due to restrictions on going out. If the physical performance and motor imagery before and after the restrictions on going out were measured in the same group, it would have been possible to clarify the influence of restrictions on going out on motor imagery in more detail. Second, iTUG was used to assess motor imagery in this study, following previous studies [18,19,20]. However, this does not mean that only an iTUG assessment could explain all of the motor imagery. Third, as objective physical activity was not investigated, it cannot be said that the amount of physical activity decreased “absolutely” just because people were forced not to go out. Fourth, this study had a small sample and was not targeted across multiple regions or cities. Finally, it is possible that it was easier for subjects to understand the measurement method because they were all medical university students.

Therefore, it is necessary to objectively measure the amount of physical activity using wearable devices in the future. In addition, in order to assess motor imagery, it is necessary to explore proven assessment tools other than iTUG and combinations of such assessment tools. Regarding the selection of subjects, it is necessary to perform research on people living in different settings, such as people who are not university students (those who are already working).

Even today, infection caused by COVID-19 has not completely subsided. In the future, instead of restricting going out, it will be necessary for governments to carefully con-sider public health policy, otherwise, it will cause people’s physical activity to decrease. It is necessary to devise exercises that can be done at home even if restrictions on going out are required due to the spread of another infectious disease, and to verify their effectiveness.

## 5. Conclusions

This study revealed that it may be difficult for medical university students to generate appropriate motor imagery, even though they are not older adults or stroke patients. It is thought that restrictions on going out (no restrictions on ADL at home) affected motor imagery. However, because the sample size was small and the number of subjects was limited, as was the demographic of subjects, we cannot be sure that ‘restrictions on going out reduce the motor imagery in all young people’. From the results of this study, as a piece of advice, it may not be possible to show the effect of training using motor imagery for those who are restricted from going out. Therefore, it is better to evaluate the appropriate motor imagery in advance for training using motor imagery.

## Figures and Tables

**Table 1:** Characteristics of subjects.

Variables		Mean (SD) or Number (%)	
		Total	
		n = 83	
Age, y		19.6	(0.6)
GPA, score		2.7	(0.5)
Height, cm		167.1	(8.7)
Weight, kg		59.6	(9.6)
BMI, kg/m^2^		21.2	(2.3)
Sex, male		43	(51.8)
Primary means of commuting to university			
	Walking	0	(0)
	Bicycle	15	(18.1)
	Motorcycle	0	(0)
	Train	68	(81.9)
	Other	0	(0)

Abbreviations: GPA, grade point average; BMI, body mass index.

**Table 2:** Comparison between the R-group and NR-group.

Variables		Mean (SD) or Number (%)					
		R-Group		NR-Group		*p* Value	Effect Size
		n = 42		n = 41			(*r*,*φ*)
Age, y		19.6	(0.5)	19.6	(0.6)	n.s.	0.03
GPA, score		2.8	(0.3)	2.7	(0.5)	n.s.	0.13
Height, cm		165.4	(7.8)	167.1	(8.7)	n.s.	0.10
Weight, kg		58.6	(10.6)	59.6	(9.6)	n.s.	0.05
BMI, kg/m^2^		21.3	(2.7)	21.2	(2.3)	n.s.	0.02
Sex, male		20	(47.6)	23	(56.1)	n.s.	0.09
Primary means of commuting to university							
	Walking	0	(0)	0	(0)	n.s.	0.04
	Bicycle	7	(16.7)	8	(19.5)		
	Motorcycle	0	(0)	0	(0)		
	Train	35	(83.3)	33	(80.5)		
	Other	0	(0)	0	(0)		
MWS, m/s		2.3	(0.4)	2.1	(0.3)	n.s.	0.11
TUG, s		7.3	(1.6)	7.6	(1.2)	n.s.	0.11
iTUG, s		5.8	(1.4)	7.4	(1.6)	<0.001	0.48
ΔTUG, %		15.4	(16.3)	1.7	(10.9)	<0.001	0.43
FRT, cm		41.7	(6.2)	39.8	(5.7)	n.s.	0.16
Grasp strength, kg		32.9	(8.7)	36.4	(9.1)	n.s.	0.19
5CS, s		5.7	(1.0)	5.5	(0.9)	n.s.	0.04

Abbreviations: R-group, restriction of outings group; NR-group, non-restriction of outings group; GPA, grade point average; BMI, body mass index; MWS, maximum walking speed; TUG, Timed Up and Go test; iTUG, imagined Timed Up and Go test; FRT, functional reach test; 5CS, 5 chair stand; n.s, not significant. Calculated from the formula: delta time (ΔTUG) = [(TUG − iTUG)/(TUG + iTUG)/2] × 100.

**Table 3:** Main effects and interactions of restriction of outings and sex on ΔTUG.

	Number (%)				Main Effect			Interaction Effect		
Variable	R-Group		NR-Group		(Restriction of Outings)			(Sex × Restriction of Outings)		
Sex	n = 42		n = 41		*F*	*p* Value	Effect Size (*η*^2^)	*F*	*p* Value	Effect Size (*η*^2^)
male	20	(47.6)	23	(56.1)	18.95	<0.001	0.19	0.168	0.683	0.002
female	22	(52.4)	18	(43.9)						

Abbreviations: R-group, restriction of outings group; NR-group, non-restriction of outings group.

## Data Availability

Not applicable.
